# Arthroscopic Treatment of a “Bucket-Handle Like Tear” Lesion of the Medial Meniscus

**DOI:** 10.7759/cureus.22830

**Published:** 2022-03-03

**Authors:** Antonis Kouzelis, Konstantina Solou, Andreas Panagopoulos, Zinon Kokkalis, John Gliatis

**Affiliations:** 1 Orthopaedic Department, Medical School, University of Patras, Patras, GRC; 2 Orthopaedic Surgery Department, Medical School, University of Patras, Patras, GRC; 3 Orthopaedics Department, School of Health Rehabilitation Sciences, University of Patras, Patras, GRC

**Keywords:** surgical repair, knee anatomy, arthroscopic resection, bucket handle tear, meniscus surgery

## Abstract

Menisci are involved in providing shock absorption, knee stability, and load transfer. Age, tear pattern, location, size and extent, repair time and technique, and patient habits are among various factors that affect meniscal healing. Meniscus repair has become the procedure of choice for the treatment of meniscal tears. However, treatment of meniscal tears in patients over 40 years of age is still debatable. Rare patterns of lesions have been described in the literature. We report a zone 2, partial thickness, “bucket-handle like tear” medial meniscal lesion with two attached ends in a 48-year-old male patient with persistent symptoms after six months of conservative treatment. Arthroscopic excision and debridement were performed. At a six-month follow-up, the patient regained 90% of his functional capacity.

## Introduction

Among the most common knee injuries, meniscal tears affect 60-70 per 100,000 of the population with an incidence of 12% to 14% [1]. A bucket handles meniscal lesion is by definition a longitudinal and full-thickness tear where the inner part migrates centrally, which is more commonly located at the peripheral zone of the meniscus. Bucket-handle tears occur seven times more often at the medial meniscus than the lateral one [2]. Longitudinal meniscal tears usually affect the posterior horns and the peripheral third of the meniscus [3].

Based on the blood supply, from medial and lateral genicular arteries, menisci are divided from outer to inner into three zones: red-red, red-white, and white-white zones. It has been proved that the more medially the tear is located, the less likely it is to heal [4]. 

Many patterns of non-typical meniscal lesions, along with their treatment, have been described. To our knowledge, there is no report in the published literature of a zone 2, partial thickness, “bucket-handle like tear” lesion with two attached ends. The present study describes a case of a 48-year-old male patient with a zone 2, partial thickness “bucket-handle like tear” meniscal lesion with two attached ends, treated with partial meniscectomy and arthroscopic debridement.

## Case presentation

**Figure 1:**
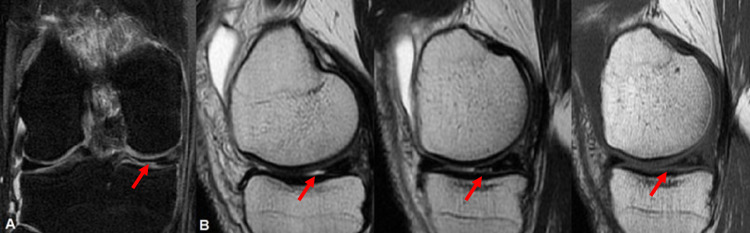
(A) MRI T2 coronal image shows partial-thickness rupture of zone 2 of the medial meniscus (red arrow). (B) MRI T1 sagittal consecutive images show the same rupture of zone 2 of the medial meniscus (red arrows).

The reported patient, a 48-year-old fit man with no underlying health issues, presented to our care with acute right knee pain, which occurred during work (gardening). Additionally, he reported locking-like symptoms during walking and kneeling which resolved automatically. In physical examination, the pain was present during the full range of motion. McMurray and Thessaly tests were positive while Pivot Shift and Anterior Drawer tests were negative. The magnetic resonance imaging (MRI) revealed signs of partial medial meniscal rupture, increased magnetic signal in the medial collateral ligament, and signs of liquid in the medial compartment of the knee (Figures 1A, 1B). Initially, the patient was treated conservatively with partial weight-bearing, non-steroid anti-inflammatory drugs, and physiotherapy.

**Figure 2:**
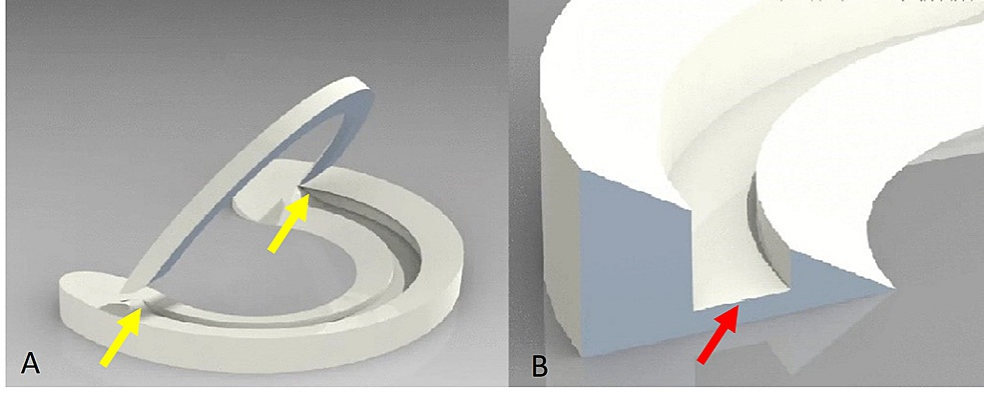
Partial in two dimensions - length and height - “bucket-handle like tear” lesion. (A) In length (yellow arrows). (B) In height (red arrow).

Six months later, pain and locking were still present, so electively the patient underwent a partial meniscectomy of the medial meniscus tear under general anesthesia by a senior orthopedic consultant. The arthroscopic finding was a horizontal, partial thickness, “bucket-handle like tear” lesion of the red-white zone of the medial meniscus (Figures 2A, 2B).

**Figure 3:**
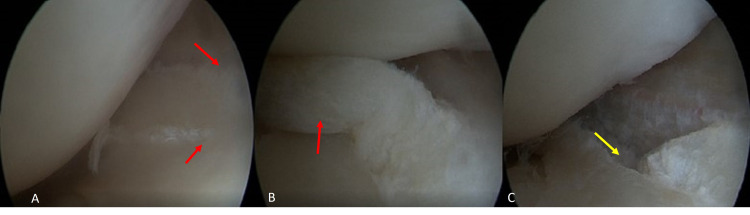
Arthroscopic excision and debridement. (A) The distal end of “bucket-handle like tear” (red arrows). (B) The central flap of meniscal tear (red arrow). (C) After the excision of the free flap the remaining body of the meniscus was intact (yellow arrow).

The tear was released from both proximal and distal ends, excised, and refreshed without any further interventions since the body of the remaining meniscus was intact (Figures 3A-3C) Anterior and posterior cruciate ligaments, lateral meniscus and chondral areas of the knee were intact.

The patient was discharged from the hospital the next day in good general condition. There were no complications and the patient started full weight the second postoperative week. On a six-month follow-up, the patient had an excellent functional outcome with a Lysholm Knee score of 90%.

## Discussion

Literature research has not revealed any similar cases of such meniscal tears. The menisci play a significant role in knee biomechanics. As a result, any pathology or the absence of the menisci can affect knee function. Studies on longitudinal tears located at the red-white zone demonstrated failure rates between 0% and 38% for the inside-out suture repair technique [5], while the failure rates for the all-inside technique were between 0% and 80% [6]. Consequently, the outcome of repairing meniscal lesions of the red-white zone is still under debate. A systematic review analyzing the clinical outcome after red-white zone meniscal tear repair showed 83% of total healing after arthroscopy, while pooled failure rates fluctuate between 22.3% and 24.3% [7].

In our case, due to the patient’s age, we initially proposed conservative treatment to avoid post-operative problems and complications after suturing in a degenerative meniscal environment. However, there were serious complaints at the six-month follow-up and a knee arthroscopy was decided. The rest of the meniscus was found to be intact and thus was preserved. Hence, no meniscectomy but only excision of the meniscal flap and freshening of the remaining area was performed in order to enhance tissue healing. Since the tear was partial in two dimensions - length and height, we had to convert it to a full-thickness tear in order to suture it. Degenerative meniscus and location of the lesion at the red-white zone made this choice less favorable.

## Conclusions

A partial, in two dimensions - length and height - “bucket-handle like tear” lesion of the medial meniscus is a very real entity that has never been described before. Reporting and identifying this kind of lesion in middle-aged patients help choose the best treatment option. Arthroscopic excision and debridement can provide fine midterm postoperative results.
